# Double bundle ACL reconstruction leads to better restoration of knee laxity and subjective outcomes than single bundle ACL reconstruction

**DOI:** 10.1007/s00167-021-06744-z

**Published:** 2021-09-30

**Authors:** Arttu Seppänen, Piia Suomalainen, Heini Huhtala, Heikki Mäenpää, Tommi Kiekara, Timo Järvelä

**Affiliations:** 1 Faculty of Medicine and Health Technology, Tampere University, Arvo Ylpön katu 34, 33520 Tampere, Finland; 2 Tampere University Hospital Orthopaedics Trauma Unit, Tampere, Finland; 3 Faculty of Social Sciences, Tampere University, Tampere, Finland; 4 Department of Orthopaedics, Tampere University Hospital, Faculty of Medicine and Health Technology, University of Tampere, Tampere, Finland; 5 Medical Imaging Center, Tampere University Hospital, Faculty of Medicine and Health Technology, Tampere University, Tampere, Finland; 6 Sports Medicine and Arthroscopic Center, Hospital Mehiläinen, Tampere, Finland

**Keywords:** Anterior cruciate ligament, ACL, Reconstruction, Meta-analysis, Single bundle, Double bundle

## Abstract

**Purpose:**

The purpose of this meta-analysis is to compare arthroscopic single bundle (SB) and double bundle (DB) anterior cruciate ligament (ACL) reconstructions in the light of all available randomised controlled trials (RCTs). A meta-analysis of this well-researched topic was performed and subgroup analyses of the medial portal (MP) technique and the transtibial technique (TT) were added as a new idea. The hypothesis was that the DB technique is superior to the SB technique also in subgroup analyses of the MP and TT techniques.

**Methods:**

Instructions of the PRISMA checklist were followed. Systematic literature search from electronic databases, including PubMed, Cochrane library and Scopus was performed to find RCTs that compared the SB and DB techniques. Nine outcomes were used to compare these two techniques. Each study was assessed according to the Cochrane Collaboration’s risk of bias tool and three subgroup analyses (minimum 2-years’ follow-up, TT technique and MP technique) were performed.

**Results:**

A total of 40 studies were included in this meta-analysis. When analysing all the included studies, the DB technique was superior to the SB technique in KT-1000/2000 evaluation (*p* < 0.01), IKDC subjective evaluation (*p* < 0.05), Lysholm scores (*p* = 0.02), pivot shift (*p* < 0.01) and IKDC objective evaluation (*p* = 0.02). Similar results were also found in the subgroup analyses of minimum 2-years’ follow-up and the TT technique. However, there were no differences between the two techniques in a subgroup analysis of the MP technique.

**Conclusion:**

Generally, DB ACL reconstruction leads to better restoration of knee laxity and subjective outcomes than SB ACL reconstruction. The subgroup analysis of the MP technique revealed that surgeons can achieve equally as good results with both techniques when femoral tunnels are drilled through the medial portal.

**Level of evidence:**

II.

**Supplementary Information:**

The online version contains supplementary material available at 10.1007/s00167-021-06744-z.

## Introduction

The anterior cruciate ligament is an important stabilising structure of the knee that prevents anterior tibial translation and additionally maintains the rotatory stability of the knee. Nowadays, it is generally accepted that the ACL comprises two fibres that work in different ways: the anteromedial (AM) fibre and the posterolateral (PL) fibre. Although both fibres provide anterior and rotatory stability to the knee, the amount of stability provided depends on the amount of knee flexion. The AM part of the ACL prevents anterior tibial translation, especially when the knee is flexed between 60 and 90 degrees. Conversely, the PL part of the ACL sustains the anterior and rotatory stability of the knee when the knee is extended or nearly extended [16, 59].

ACL reconstruction is a common surgical procedure that aims to restore the stability of the knee. Over many decades, the single-bundle (SB) technique has been the gold standard technique for ACL reconstruction. However, the double-bundle (DB) technique better mimics the original anatomy of the ACL than the SB technique, as both the AM and PL parts of the ACL are reconstructed in the DB technique. The aim of the DB technique is to produce more rotatory and anterior stability to the knee compared to the SB technique, especially in lower flexion angles [15, 19, 20].

Despite numerous RCTs and meta-analyses, there is still no consensus as to which of the techniques is superior for ACL reconstruction. Eleven previous meta-analyses have found significant differences in at least one outcome between the two techniques, favouring the DB technique [9, 11, 12, 14, 29, 31, 32, 36, 50, 54, 61]. Those outcomes favouring the DB technique were most commonly related to restoration of knee laxity. Two meta-analyses [10, 13] found the techniques to be equally effective. However, none of the previous meta-analyses favoured the SB technique in any of the outcomes.

The purpose of this meta-analysis is therefore to compare these two surgical methods in the light of current information based on all the available RCTs. The hypothesis was that the DB technique is superior to the SB technique also in subgroup analyses of the MP technique and the TT technique.

## Materials and methods

### Literature search

PubMed, the Cochrane library and Scopus were searched from inception to March 2020. Before starting the statistical process, a further search was performed in August 2020 to ensure that no new articles had been subsequently published. The following strategy for the literature search was used: (ACL or anterior cruciate ligament) AND (DB or double bundle or SB or single bundle) AND (RCT or randomized or randomised).

### Inclusion and exclusion criteria

The inclusion criteria were as follows: (1) primary arthroscopic ACL reconstruction, (2) English language, (3) human patient population, (4) RCT study design, (5) study compared SB and DB techniques, (6) follow-up period of at least 1 year, (7) all graft types, (8) all fixation techniques, (9) all femoral drilling techniques, (10) study included at least one of the nine outcomes mentioned under the heading “Outcomes”.

The exclusion criteria were the following: (1) ongoing studies, (2) not RCTs or not SB versus DB studies, (3) full texts not available (abstracts, letters, case reports), (4) study included only irrelevant outcomes, (5) allografts used, (6) extra articular tenodesis used.

### Data extraction

**Fig. 1:**
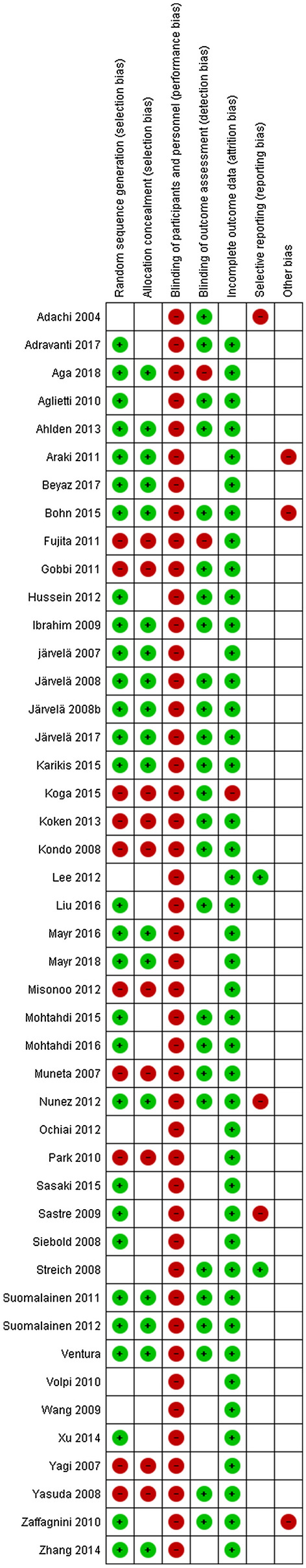
Risk of bias summary. *Green* low risk of bias, *Red* high risk of bias, *empty* unknown risk of bias

Data were extracted independently by the first author (AS). Primary information on the included studies, such as author, year of publication, level of evidence, number of patients, length of follow-up, types of graft used, the femoral drilling technique used and the fixation types used, were extracted (Table S1 in ESM). The risk of bias for each study is presented in Fig. 1.

### Outcomes

In total, nine outcomes (KT-1000/2000, IKDC subjective evaluation, Lysholm score, Tegner score, pivot shift test, IKDC objective evaluation, Lachman test, graft failures and OA changes) were used to compare the SB and DB techniques. In the included studies, the treated knees were compared to the contralateral knees in pivot shift, Lachman test and KT evaluation. Pivot shift and Lachman test results were classified from 0 to 3 across the eligibility data. In this meta-analysis, values from 1 to 3 were considered to be positive results and 0 a negative result. In the IKDC objective evaluation, grade A was considered to be a normal result and grades B, C and D as abnormal results. Osteoarthritic (OA) findings were evaluated using weight-bearing radiography and Kellgren-Lawrence (KL) classification: grade 0 normal, grade 1 minimal or uncertain degeneration and grades 2–4 definite OA. KT-1000 and KT-2000 values were measured at 20–30 degrees of knee flexion using 134 N or manual maximum force and side-to-side differences between both knees were recorded.

### Risk of bias

**Fig. 2:**
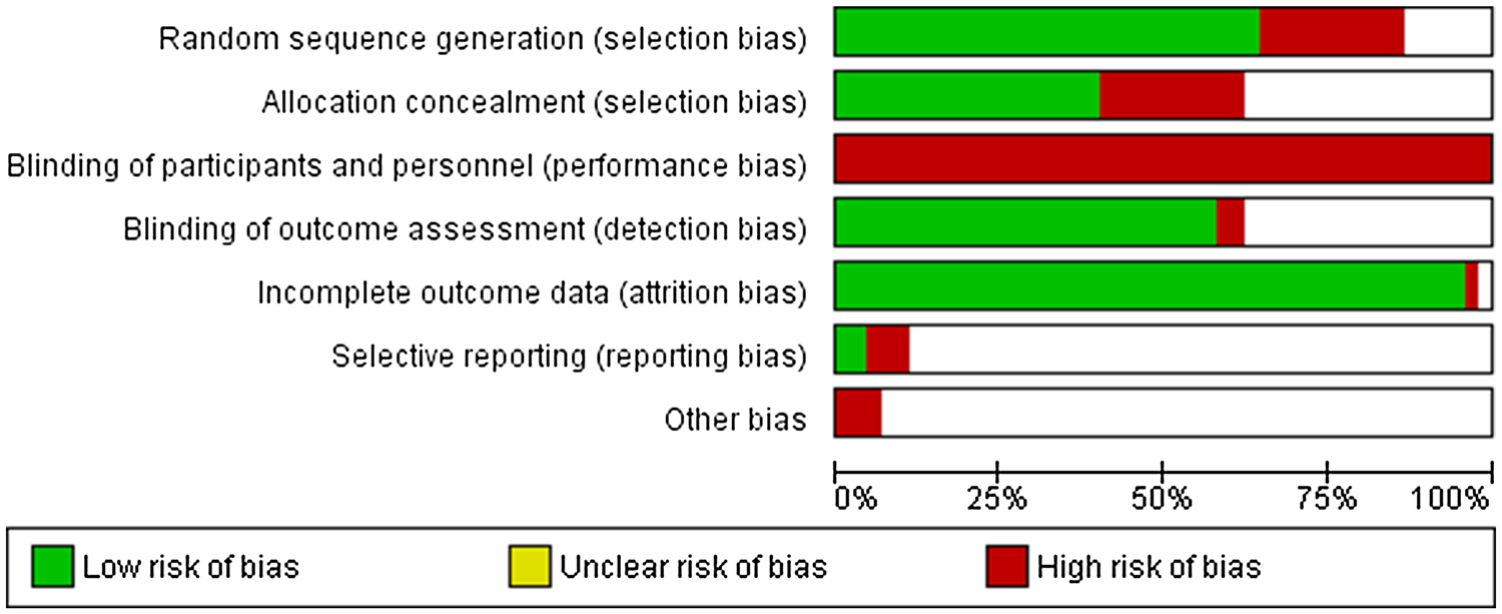
Graphic representation of biases

Each study was assessed according to Cochrane Collaboration’s risk of bias tool. The risk of bias tool includes the following six divisions: random sequence generation, allocation concealment, blinding of patients and personnel, incomplete outcome data, selective reporting and other bias. All divisions were evaluated as low risk, unclear risk or high risk of bias. The risk of bias across the initial studies are presented in Fig. 1 (summary) and Fig. 2 (graph).

### Statistical analysis

Continuous outcomes (KT-1000/2000, IKDC subjective, Lysholm score, Tegner score) were assessed using weighted mean difference (WMD). Dichotomous outcomes (pivot shift test, IKDC objective, Lachman test, graft failures, OA changes) were assessed using risk ratio (RR), and 95% confidence intervals (CI) were calculated. The Dersimonian and Laird random-effects model was used for each outcome to combine all initial studies. Statistical heterogeneity was evaluated with both the *I*^2^ test and the Chi-squared test. Statistically significant heterogeneity was present if the *p*-value of the Chi-squared test was < 0.1. *I*^2^ test results were analysed as follows: 0–40% might not be important heterogeneity, 30–60% moderate heterogeneity, 50–90% substantial heterogeneity and 75–100% considerable heterogeneity [18].

## Results

**Fig. 3:**
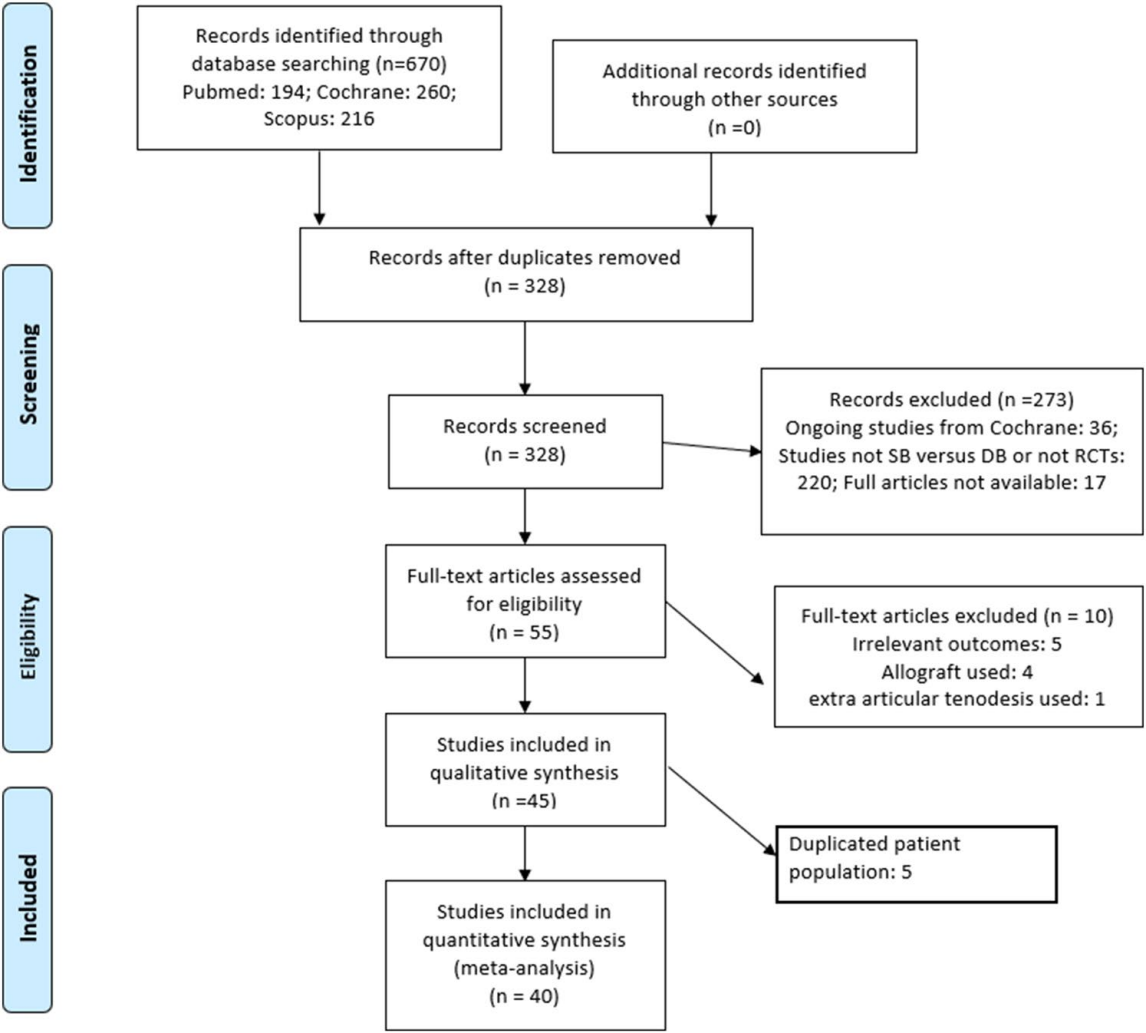
Flow diagram of study selection process

A total of 670 studies were identified from PubMed (194), the Cochrane library (260) and Scopus (216). After duplicated studies from different sources were removed, 328 articles were manually screened based on titles, abstracts and full texts. During the screening process, 273 articles were excluded. The excluded articles were either missing full texts (17), were ongoing studies (36) or not RCTs, or were not SB versus DB studies (220). Full texts were assessed to find eligibility data, and 8 articles were excluded because allografts were used (4) or outcomes were irrelevant for the meta-analysis (5). Finally, 45 RCTs were included in this study. Of these, five studies [22–24, 48, 49] were later excluded from the quantitative analysis due to duplicated patient populations. However, although Muneta et al. [40] and Koga et al. [26] shared the same patient population, both studies were included in the quantitative analysis, as they used different outcomes. The same principle was applied to two studies by Mayr et al. [34, 35] and Mohtahdi et al. [38, 39], which also shared the same patient population. The strategy to find eligibility data is described in Fig. 3. The years of publication of the eligibility data ranged from 2004 to 2018. The shortest follow-up time was 1 year and the longest 10 years.

### Subjective outcomes (all studies analysed)

**Fig. 4:**
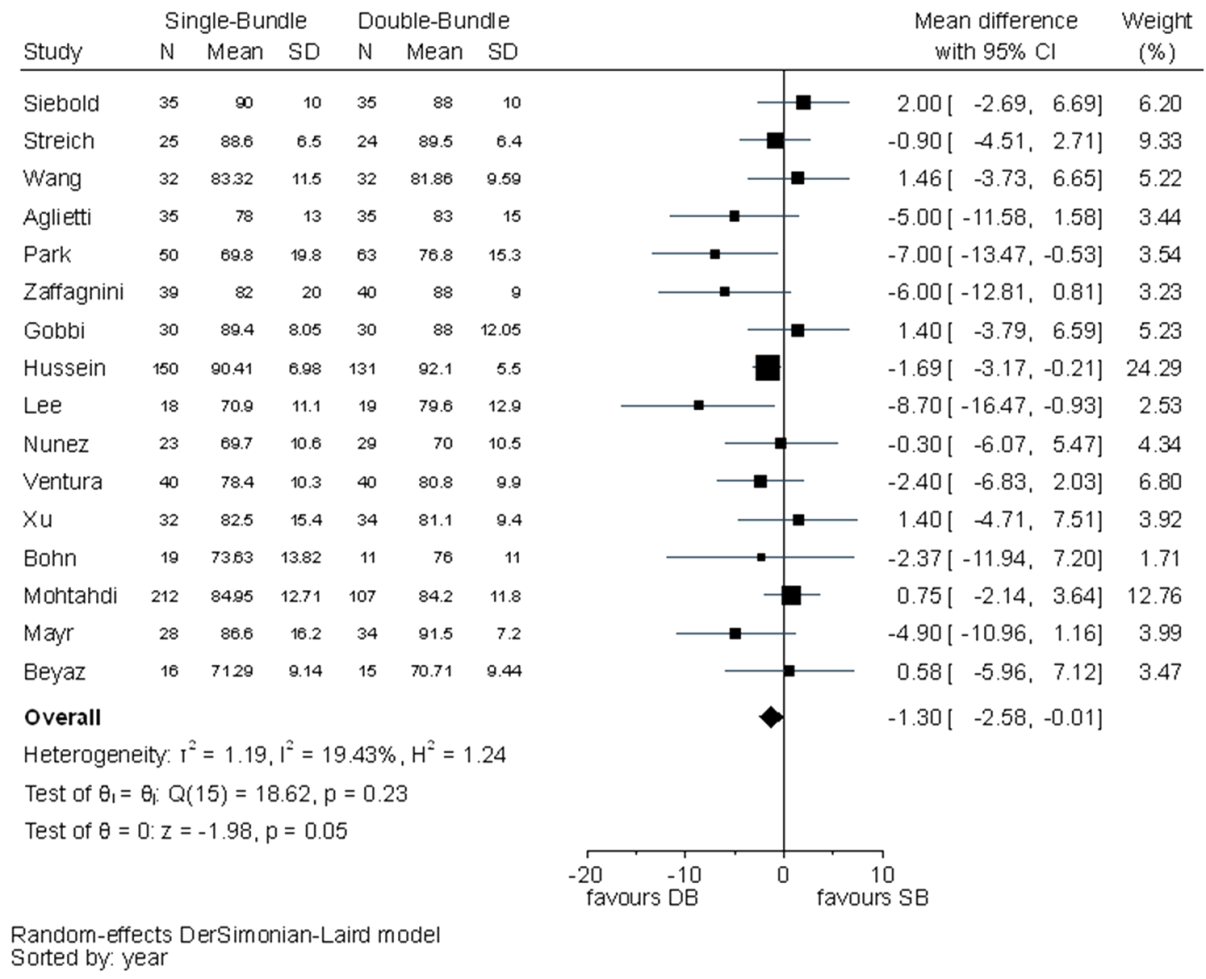
Pooled results of IKDC subjective evaluation. *SD* standard deviation, *CI* confidence interval, *SB* single bundle, *DB* double bundle

**Fig. 5:**
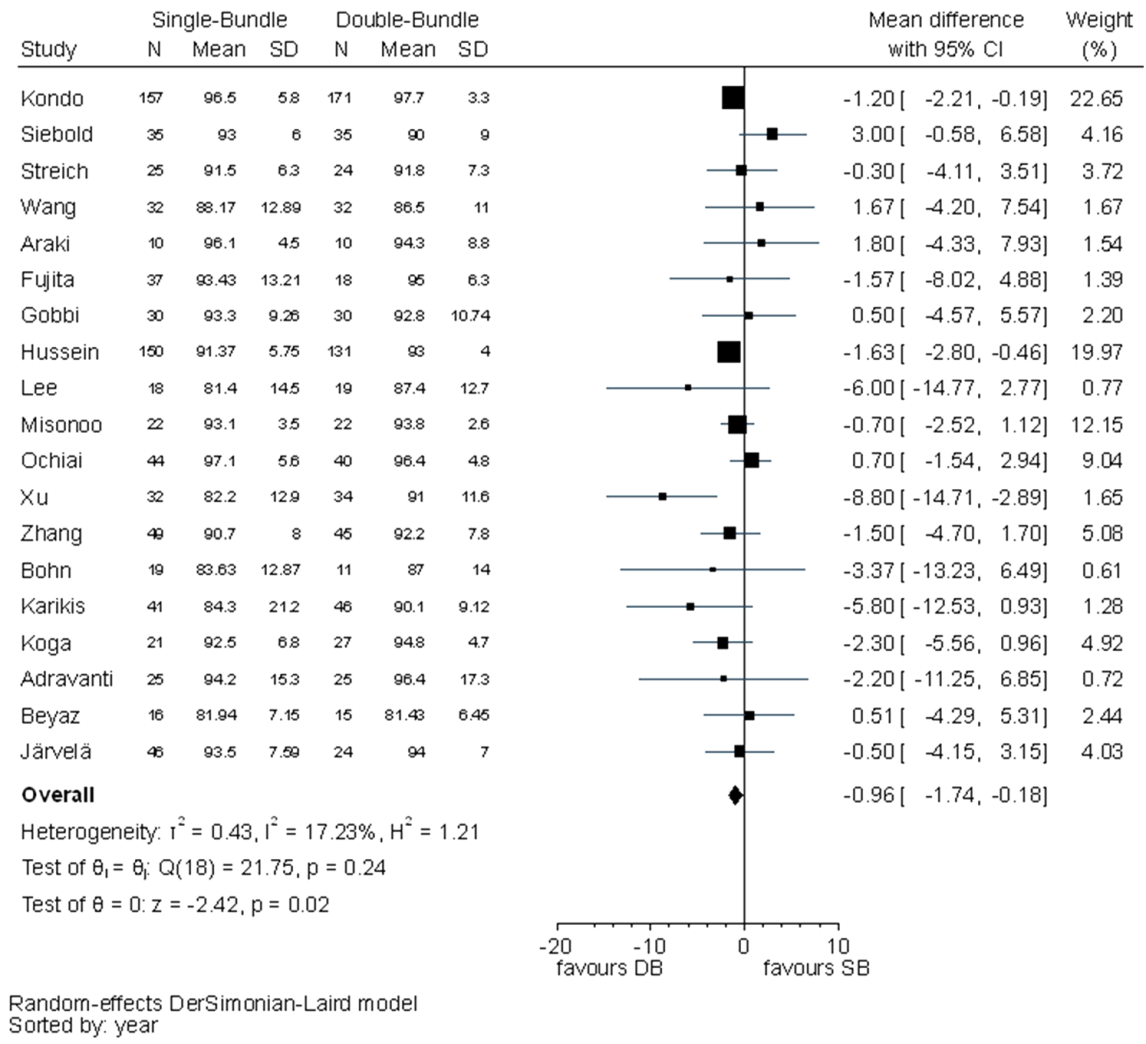
Pooled results of Lysholm scores. *SD* standard deviation, *CI* confidence interval, *SB* single bundle, *DB* double bundle

Statistically significant differences between the SB and DB techniques were found in IKDC subjective evaluation (MD − 1.30, 95% CI − 2.58 to − 0.01) and Lysholm scores (MD − 0.96, 95% CI − 1.74 to − 0.18) favouring the DB technique (Figs. 4, 5). The *I*^2^ test showed no important heterogeneity in both outcomes.

**Fig. 6:**
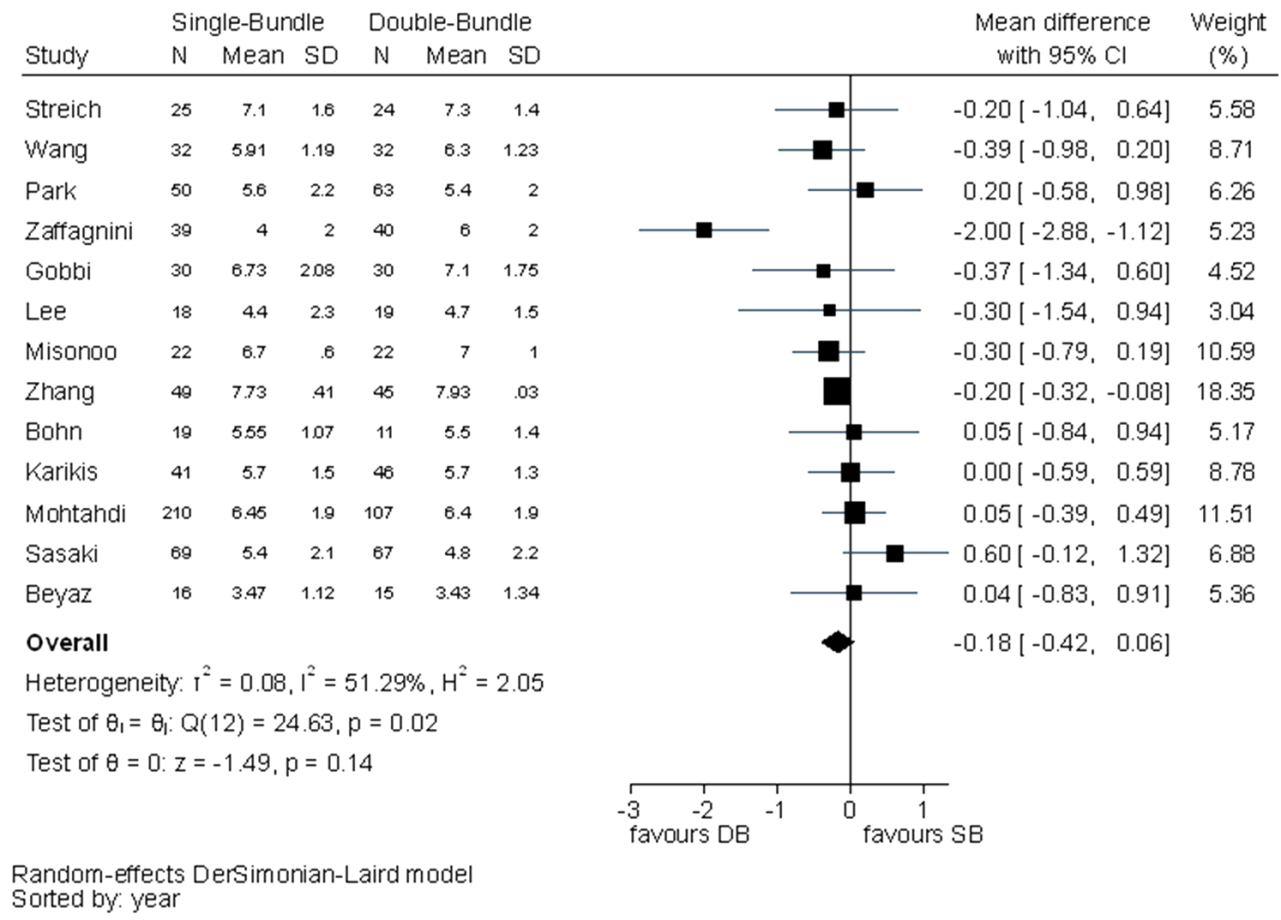
Pooled results of Tegner scores. *SD* standard deviation, *CI* confidence interval, *SB* single bundle, *DB* double bundle

No statistically significant difference was found in Tegner scores between the surgical techniques (MD − 0.18, 95% CI − 0.42–0.06). The *I*^2^ test showed moderate to substantial heterogeneity (Fig. 6).

### Objective outcomes (all studies analysed)

**Fig. 7:**
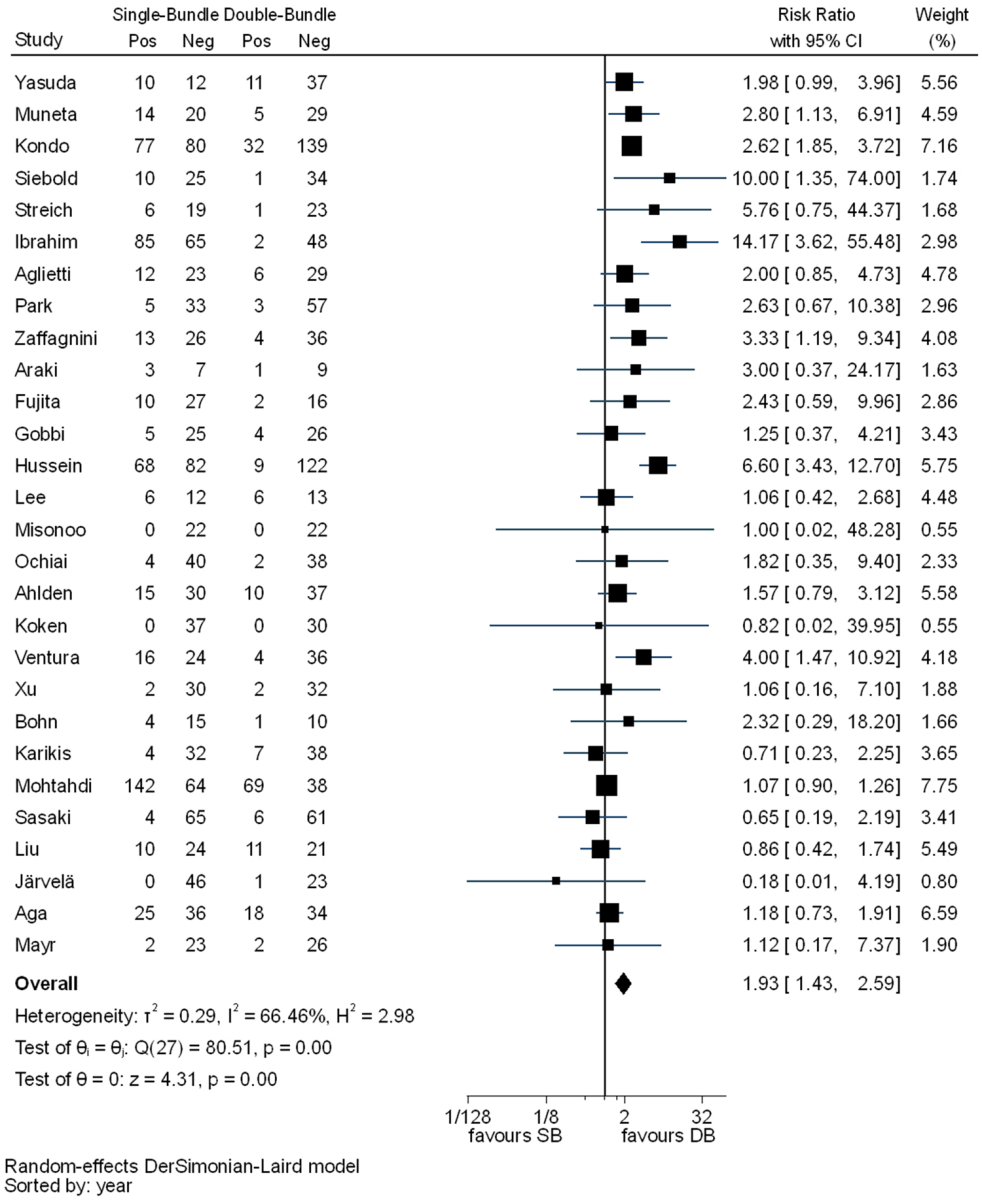
Pooled results of pivot shift. *Pos* positive, *Neg* negative, *CI* confidence interval, *SB* single bundle, *DB* double bundle

**Fig. 8:**
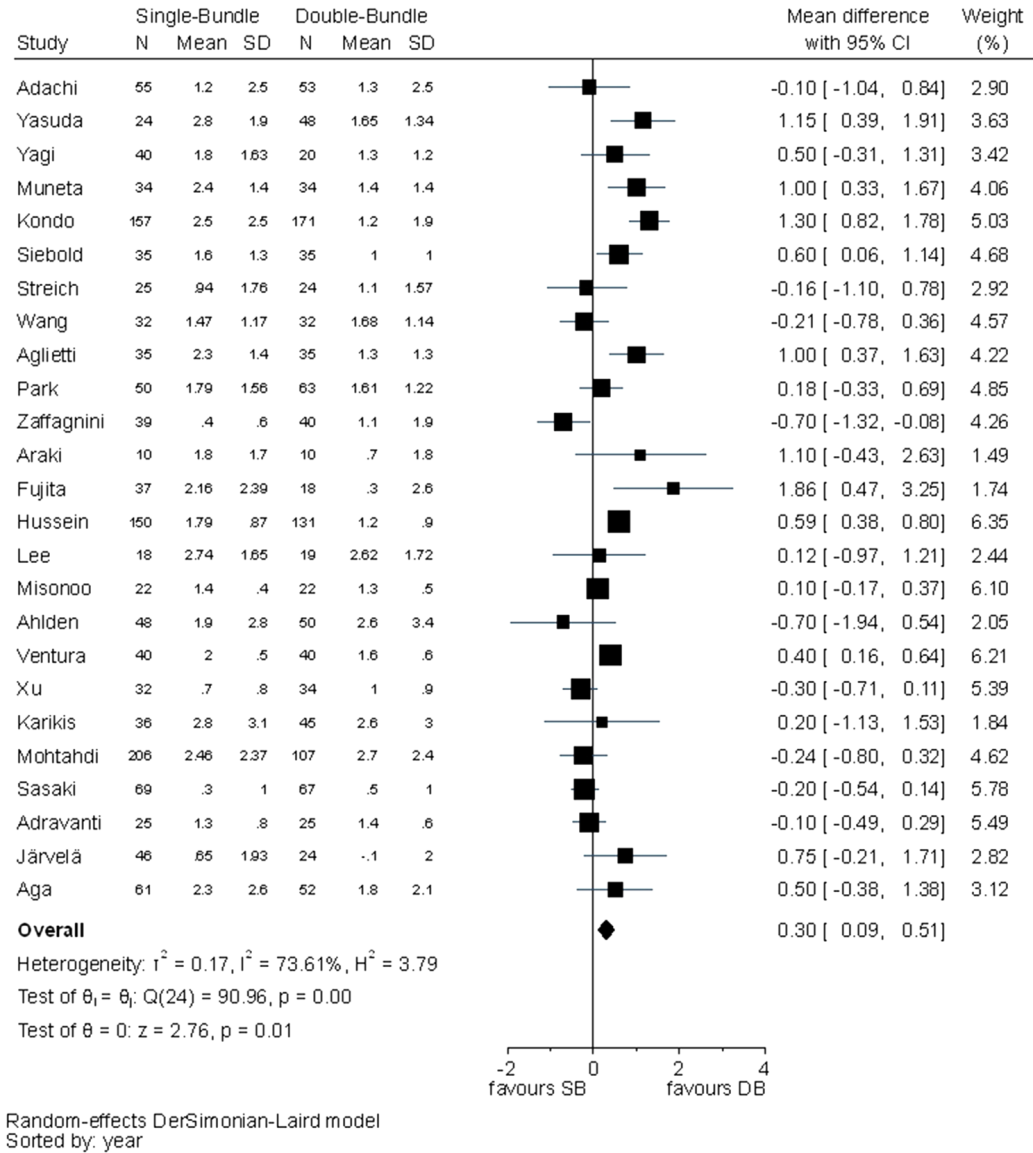
Pooled results of KT-1000/2000 arthrometer. *SD* standard deviation, *CI* confidence interval, *SB* single bundle, *DB* double bundle

**Fig. 9:**
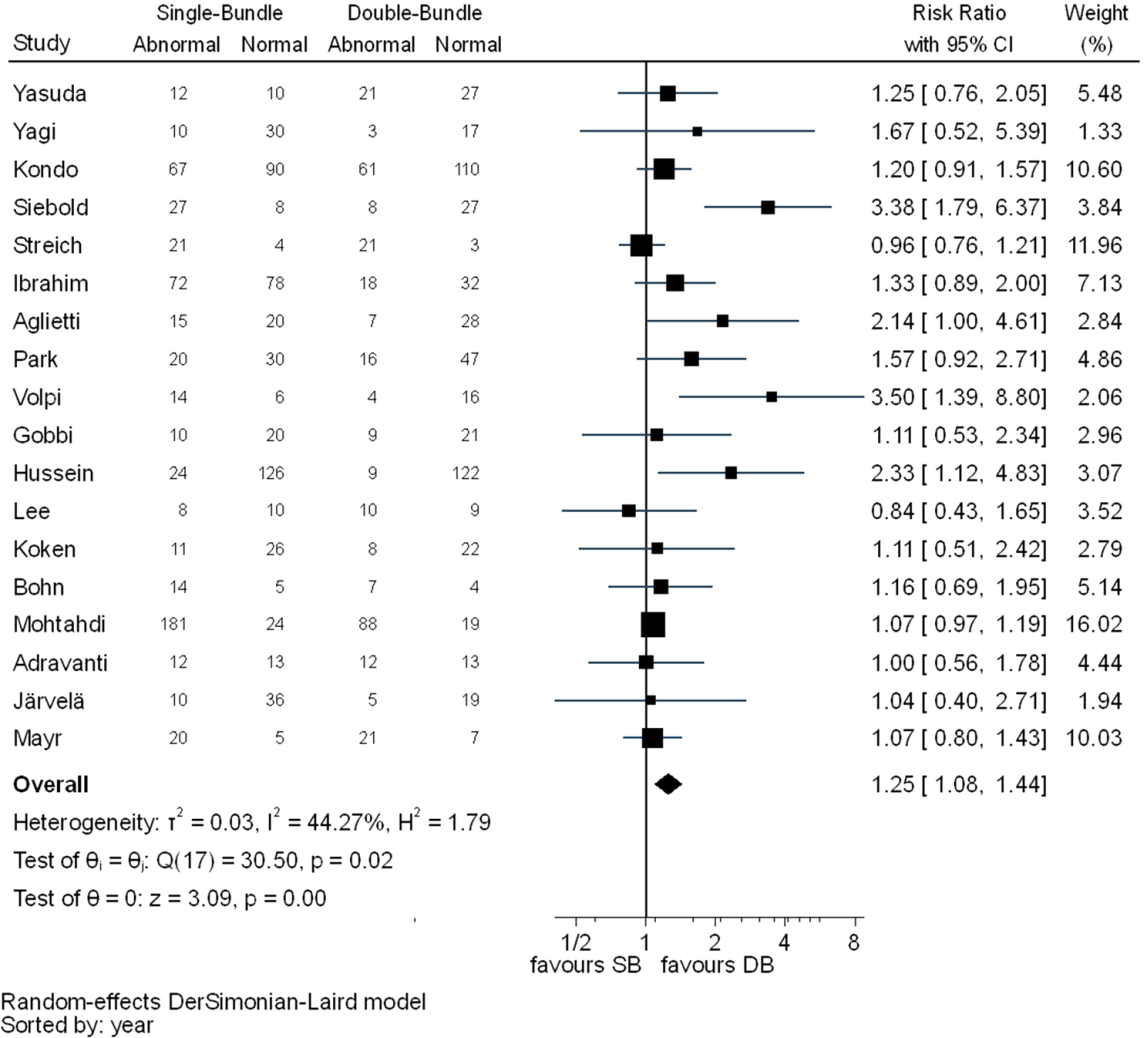
Pooled results of IKDC objective grades. *N* normal, *AN* abnormal, *CI* confidence interval, *SB* single bundle, *DB* double bundle

Statistically significant differences between the two techniques were detected in pivot shift test (RR 1.93, 95% CI 1.43–2.59), KT-1000/2000 (MD 0.30, 95% CI 0.09–0.51) and IKDC objective evaluation (RR 1.25, 95% CI 1.08–1.44) favouring the DB technique. In pivot shift and KT-1000/2000, *I*^2^ test showed substantial heterogeneity (Figs. 7, 8). For IKDC objective evaluation, *I*^2^ test showed moderate heterogeneity (Fig. 9).

**Fig. 10:**
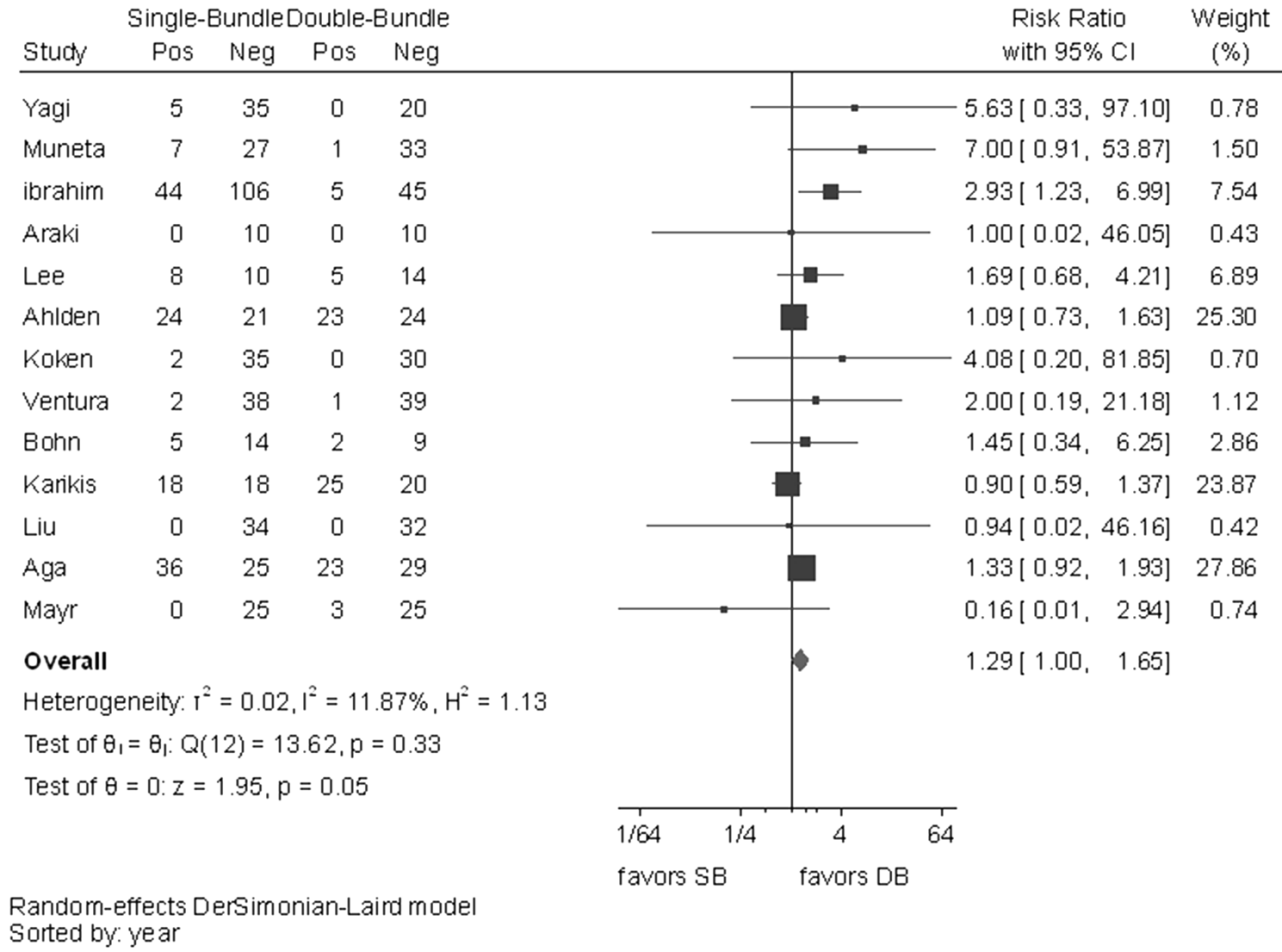
Pooled results of Lachman test. *Pos* positive, *Neg* negative, *CI* confidence interval, *SB* single bundle, *DB* double bundle

**Fig. 11:**
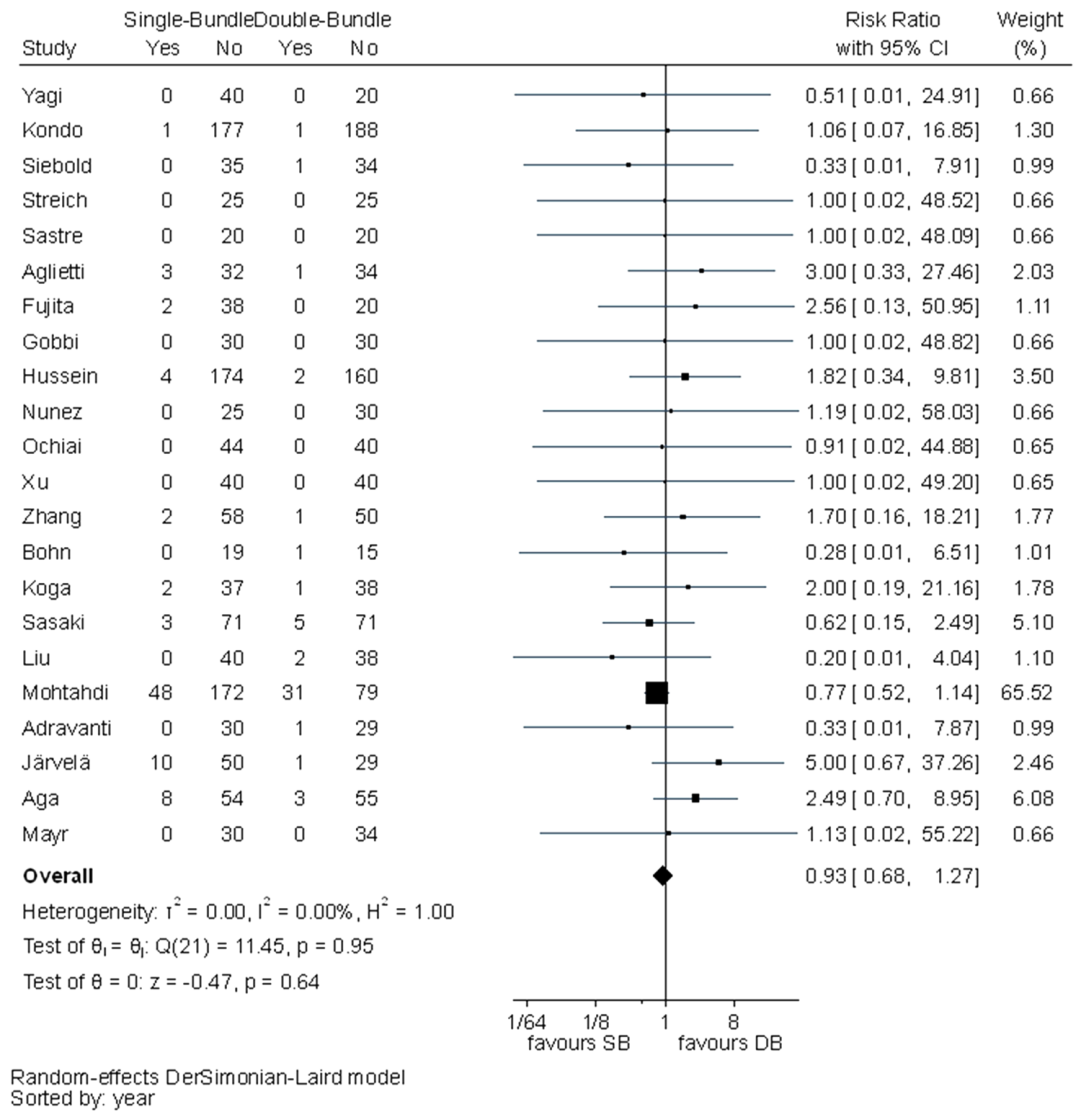
Pooled results of graft failures. *CI* confidence interval, *SB* single bundle, *DB* double bundle

**Fig. 12:**
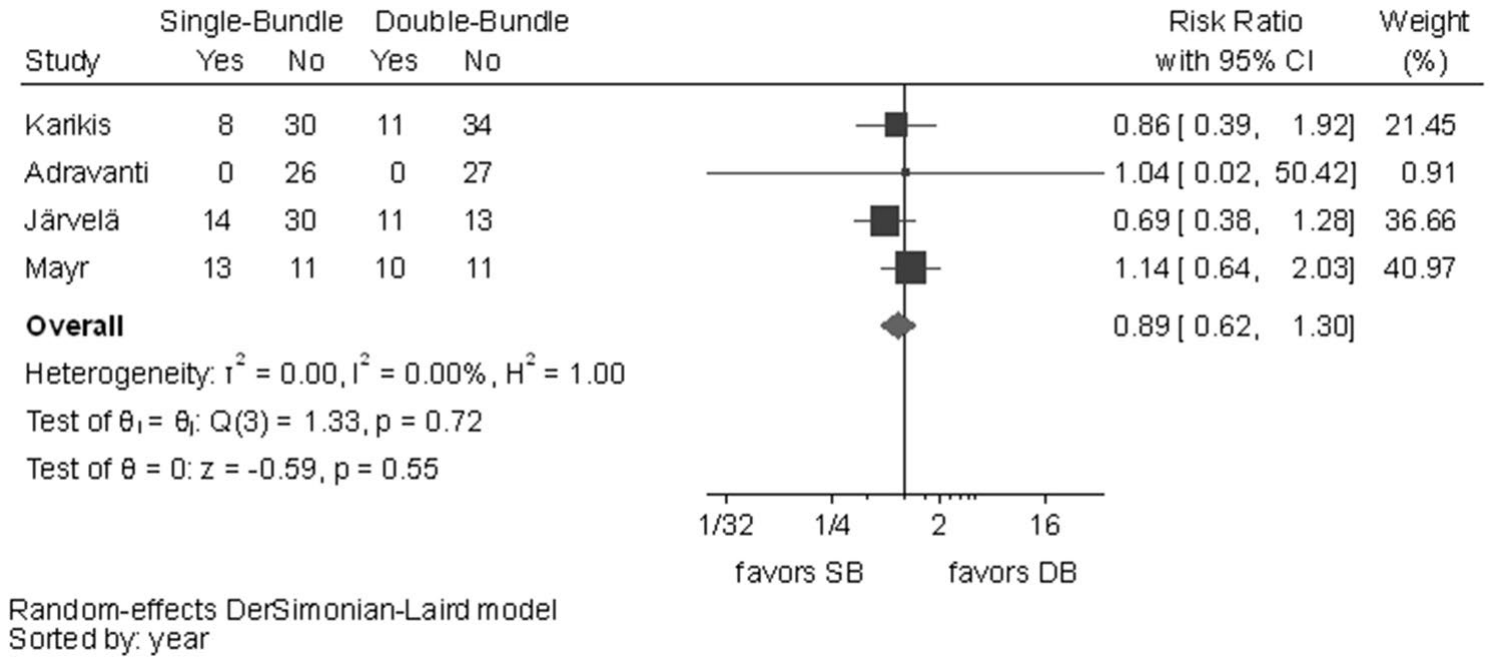
Pooled results of OA changes. *CI* confidence interval, *SB* single bundle, *DB* double bundle

No statistically significant difference was observed between the surgical techniques in Lachman test (RR 1.29, 95% CI 1.00–1.65), graft failures (RR 0.93, 95% CI 0.68–1.27) or OA changes (RR 0.89, 95% CI 0.62–1.30). In all these outcomes, *I*^2^ test showed no important heterogeneity (Figs. 10, 11, 12).

### Risk of bias

Eighteen of the 45 studies (40%) met the criteria in both random sequence generation and allocation concealment. Based on the blinding of the patients and personnel, all the studies were evaluated as high risk. Outcome assessment was certainly blinded in 26 studies. Moreover, almost all studies (96%) included complete outcome data.

### Publication bias assessment

**Fig. 13:**
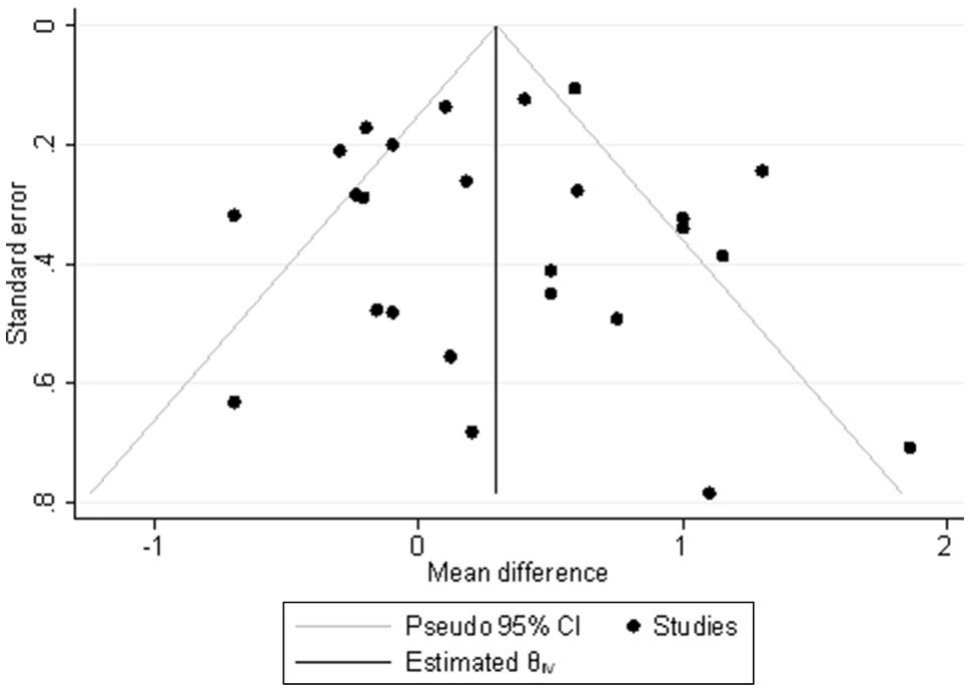
Funnel plot assessing publication bias of KT-1000/2000 results, *CI* confidence interval

A funnel plot of the KT results (Fig. 13) was used to investigate potential publication bias. The funnel plot showed asymmetry. Nine studies did not fall in the 95% confidence region because of probable heterogeneity. No studies were missing from the bottom left of the funnel plot, so there was no evidence of publication bias.

### Subgroup analysis of the minimum 2-years’ follow-up

Seven studies [6, 8, 37, 46, 52, 53, 56] were excluded from the analysis due to short follow-up period. The same outcomes (KT-1000/2000, IKDC subjective, Lysholm scores, pivot shift, IKDC objective) as before were statistically significant favouring the DB technique (Table 1).

**Table 1:** Subgroup analysis of the minimum 2-years’ follow-up

	MD or RR	95% CI	*p*-value
KT-1000/2000	MD 0.35	0.09–0.61	< 0.01*
IKDC subjective	MD − 1.84	− 3.28 to − 0.40	0.01*
Lysholm score	MD − 1.20	− 1.85 to − 0.55	< 0.01*
Tegner score	MD − 0.16	− 0.48–0.15	n.s
Pivot shift	RR 1.88	1.37–2.57	< 0.01*
IKDC objective	RR 1.16	1.03–1.31	0.01*
Lachman test	RR 1.31	0.97–1.77	n.s
Graft failure	RR 0.95	0.69–1.31	n.s
OA	RR 0.89	0.62–1.30	n.s

*MD* mean difference, *RR* risk ratio, *CI* confidence interval, *Statistically significant result, *n.s* not significant

Variation in length of follow-up was not a very important reason for statistical heterogeneity, as in many cases (KT-1000/2000, IKDC subjective, Tegner score, pivot shift, Lachman test) statistical heterogeneity, according to *I*^2^-test, increased when studies with short follow-up times were excluded from the analysis.

### Subgroup analysis of the TT technique

Subgroup analysis of the TT technique (Table 2) included studies with transtibial drilled femoral tunnels. A total of 17 studies were included to the analysis [1, 2, 15, 26, 28, 37, 40–43, 46, 47, 51, 52, 56, 57, 60]. The results of this subgroup analysis were similar to those of previous analyses. Statistically significant results favouring the DB technique were found in KT-1000/2000, Lysholm scores, pivot shift, IKDC objective evaluation, Tegner scores and Lachman test. Generally, the statistical heterogeneities of the outcomes were lower than when all the studies were analysed.

**Table 2:** Subgroup analysis of the TT technique

	MD or RR	95% CI	*p*-value
KT-1000/2000	MD 0.48	0.19–0.77	< 0.01*
IKDC subjective	MD − 1.30	− 3.76–1.16	n.s
Lysholm score	MD − 0.79	− 1.52 to − 0.05	0.04*
Tegner score	MD − 0.19	− 0.31 to − 0.07	< 0.01*
Pivot shift	RR 2.66	2.04–3.46	< 0.01*
IKDC objective	RR 1.43	1.07–1.93	0.02*
Lachman test	RR 5.14	1.32–19.92	0.02*
Graft failure	RR 1.12	0.42–2.94	n.s

*MD* mean difference, *RR* risk ratio, *CI* confidence interval, *Statistically significant result, *n.s* not significant

### Subgroup analysis of the MP technique

Subgroup analysis of the MP technique (Table 3) included studies where femoral tunnels were drilled from the medial portal [3, 5, 7, 8, 17, 19, 21, 25, 27, 33, 35, 45, 55, 58]. The results of this subgroup analysis differed from our previous analyses because no statistically significant differences were found between the SB and DB techniques. Statistical heterogeneities of the outcomes were generally lower than when all the studies were analysed.

**Table 3:** Subgroup analysis of the MP technique

	MD or RR	95% CI	*p*-value
KT-1000/2000	MD 0.02	− 0.42–0.46	n.s
IKDC subjective	MD − 1.39	− 2.78–0.00	n.s
Lysholm score	MD − 1.67	− 3.62–0.28	n.s
Tegner score	MD − 0.45	− 1.19–0.29	n.s
Pivot shift	RR 1.49	0.97–2.28	n.s
IKDC objective	RR 1.11	0.89–1.38	n.s
Lachman test	RR 1.12	0.89–1.40	n.s
Graft failure	RR 1.60	0.71–3.60	n.s
OA	RR 0.89	0.61–1.30	n.s

*MD* mean difference, *RR* risk ratio, *CI* confidence interval, *n.s* not significant

## Discussion

The most important finding of the present study was that the DB technique is superior to the SB technique in restoration of knee laxity and subjective outcomes when analysing all the included studies. Similar results were also found in the subgroup analyses of minimum 2-years’ follow-up and the TT technique. Subgroup analysis of the TT technique showed an increased number of differences between the two techniques compared to the situation when all the studies were analysed. It was noteworthy that no differences between the two techniques were found in the subgroup analysis of the MP technique. This is a new finding not reported in previous meta-analyses and may be of clinical significance.

ACL reconstructions are more anatomical when femoral tunnels are drilled through the medial portal because surgeons are not dependent on the location of the tibial tunnels. It seems that the SB technique benefits more from correctly positioned femoral tunnels than the DB technique because statistically significant differences between the two techniques disappeared in a subgroup analysis of the MP technique.

In general, the rotational laxity of the knee, as measured with pivot shift test, was better after DB reconstruction than SB reconstruction. According to the findings of our analysis, patients who underwent SB reconstruction had about twice the risk of a positive pivot shift result compared to DB reconstruction. Again, this finding was not identified in subgroup analysis of the MP technique. In their meta-analysis, Li et al. [31] suggested that the SB technique leads to higher rates of graft failures compared to the DB technique. This study revealed that there was the same number of graft failures in both techniques.

The study has several limitations. First, different graft types and fixation techniques were used in the initial studies. Second, there were variations in patient populations and rehabilitation protocols across the studies. Third, there might be subjective variation in the results of manual tests, such as pivot shift and Lachman test, depending on the assessor. Also, in several studies there were more than one outcome assessor. All the above-mentioned limitations and the fact that the length of follow-up ranged from 1 to 10 years have served to increase the heterogeneity of this meta-analyses and may also have led to an overestimation of our results. Fourth, a meta-analysis is only as good as its individual studies. There are several sources of bias across the initial studies, which may have had an influence on the relevance of the findings of this meta-analysis. Bias may also have contributed to an overestimation of our results. For example, deficiencies in random sequence generation and allocation concealment have increased selection bias. Ten studies [15, 17, 26–28, 37, 40, 43, 56, 57] were considered as quasi-RCTs because an inappropriate randomisation method was used. Most commonly, this was due to randomisation according to date of birth. Blinding of outcome assessment was insufficient in 18 studies, which increased detection bias.

More long-term RCTs comparing the SB and DB techniques are needed in future. Moreover, meta-analyses of this topic should focus more on long follow-up studies or studies with anatomic/nonanatomic reconstructions.

## Conclusion

In general, DB ACL reconstruction leads to better restoration of knee laxity and subjective outcomes than SB ACL reconstruction. The subgroup analysis of the MP technique revealed that surgeons can achieve equally as good results using both techniques when femoral tunnels are drilled through the medial portal.

## Supplementary Information

Below is the link to the electronic supplementary material.

Supplementary file1 (DOCX 39 kb)

## Abbreviations

DB: Double bundle
ACL: Anterior cruciate ligament
SB: Single bundle
RCT: Randomised controlled trial
TT: Transtibial
MP: Medial portal
PRISMA: Preferred reporting items for systematic reviews and meta-analyses
IKDC: International Knee Documentation Committee
AM: Anteromedial
PL: Posterolateral
OA: Osteoarthritis
KL: Kellgren–Lawrence
WMD: Weighted mean difference
MD: Mean difference
RR: Risk ratio
CI: Confidence interval
N: Normal
AN: Abnormal
pos: Positive
neg: Negative
n.s: Not significant
