# Supplementary material for: Double bundle ACL reconstruction leads to better restoration of knee laxity and subjective outcomes than single bundle ACL reconstruction
Source: Knee Surg Sports Traumatol Arthrosc. 2021 Sep 30;30(5):1795–808. doi: 10.1007/s00167-021-06744-z (PMC9033716; doi:10.1007/s00167-021-06744-z)
Supplement: Supplementary file 1 — Supplementary file1 (DOCX 39 kb) [file 167_2021_6744_MOESM1_ESM.docx]

**Table 1** Characteristics of included studies

| First author | Year | Design | Number of patients (SB:DB) | Follow-up | Implant | Drilling the femoral tunnel | Fixation | Outcomes |
| --- | --- | --- | --- | --- | --- | --- | --- | --- |
| Adachi [1] | 2004 | RCT | 55:53 | mean 32 mo | ST, GT | TT | EB, staple | KT-2000 |
| Yasuda [63] | 2006 | RCT  LoE 2 | 24:48 | min 24 mo | ST, GT | TT | EB, staple | KT-2000, pivot shift, IKDC objective |
| Järvelä [26] | 2007 | RCT | 25:30 | mean 14 mo | ST, GT | AMP | BIS | Lysholm score, pivot shift, IKDC objective, graft failures |
| Yagi [62] | 2007 | RCT  LoE 2 | 40:20 | min 12 mo | ST, GT | **DB**: AM (TT) PL (map)  **SB**: TT, map | EB, CaS, PC | KT-1000, IKDC objective, Lachman test, graft failures |
| Muneta [45] | 2007 | RCT  LoE 1 | 34:34 | mean 25 mo | ST | TT | EB, staple | KT-1000, pivot shift Lachman test |
| Järvelä [28] | 2008 | RCT  LoE 1 | 41:22 | min 24 mo | ST, GT | AMP | BIS, MIS | KT-1000, Lysholm score, pivot shift, IKDC objective, graft failures |
| Järvelä [27] | 2008 | RCT  LoE 1 | 25:35 | mean 27 mo | ST, GT | AMP | BIS | KT-1000, Lysholm score, pivot shift, IKDC objective, graft failures |
| Kondo [33] | 2008 | RCT  LoE 2 | 157:171 | min 24 mo | ST, GT | TT | EB, staple | KT-1000, pivot shift, Lysholm, IKDC objective, graft failures |
| Siebold [52] | 2008 | RCT  LoE 1 | 35:35 | mean 19 mo | ST, GT | **DB**: AM (TT) PL (AAMP)  **SB**: TT | EB, BIS | KT-1000, Lysholm score, IKDC subjective, pivot shift, IKDC objetive, graft failures |
| Streich [53] | 2008 | RCT | 25:24 | SB: mean 24.1 mo  DB: mean 23.8 mo | ST | TT | EB, suture-disc | KT-1000, Tegner score, Lysholm score, IKDC subjective, pivot shift, IKDC objective, graft failures |
| Ibrahim [24] | 2009 | RCT | 150:50 | mean 29 mo | ST, GT | **DB**: AMP  **SB**: TT | EB, BS, CS, CP | Lachman test, IKDC objective, pivot shift |
| Sastre [51] | 2009 | RCT | 20:20 | min 24 mo | ST, GT | **DB**: AM (AMP)  PL (map)  **SB**: map | EB; BIS | graft failures |
| Wang [59] | 2009 | RCT | 32:32 | SB: mean 17.7 mo  DB: mean 14.4 mo | ST, GT | **DB**: AMP  **SB**: TT | EB, BIS, staple | KT-2000, Lysholm score, Tegner score, IKDC subjective |
| Aglietti [4] | 2010 | RCT  LoE 1 | 35:35 | min 24 mo | ST, GT | OI | IS, staple | KT-1000, IKDC subjective, IKDC objective, pivot shift, graft failures |
| Park [48] | 2010 | RCT  LoE 2 | 50:63 | 24 mo | ST, GT | TT | EB, BS | KT-2000, pivot shift, IKDC objective, IKDC subjective, Tegner score |
| Volpi [58] | 2010 | RCT | 20:20 | SB: mean 31 mo  DB: mean 28 mo | ST, GT, BPTB | TT | BCP, staple, BS | IKDC objective |
| Zaffagnini [64] | 2010 | RCT | 39:40 | mean 8.6 year | ST, GT, BPTB | **DB**: MP  **SB**: AMP | IS, staple, TSK | KT-2000, IKDC subjective, Tegner score, pivot shift |
| Araki [7] | 2011 | RCT | 10:10 | SB: mean 12.0 mo  DB: mean 13.5 mo | ST, GT | **DB**: AM (TT) PL (AMP)  **SB**: map | EB, CaS | KT-1000, Lysholm score, pivot shift, Lachman test |
| Fujita [18] | 2011 | RCT  LoE 2 | 37:18 | DB: mean 33.7 mo | ST, GT | **DB**: AM (TT) PL (map)  **SB**: TT | EB, CaS | KT-1000, Lysholm score, pivot shift, graft failures |
| Suomalainen [55] | 2011 | RCT  LoE 1 | 60:61 | SB: mean 28 mo  DB: mean 26 mo | ST, GT | AMP | BIS, MIS | KT-1000, pivot shift, IKDC objective, graft failures |
| Gobbi [20] | 2012 | RCT  LoE 2 | 30:30 | mean 46.2 mo | ST | **DB**: AM (AMP) PL (OI)  **SB**: AMP | EB, BIS | IKDC subjective, Tegner score, Lysholm score, IKDC objective, pivot shift, graft failures |
| Hussein [23] | 2012 | RCT  LoE 1 | 150:131 | mean 51.15 mo | ST | **DB**: map  **SB**: map, TT | EB, BIS | KT-1000, Lysholm score, IKDC subjective, IKDC objective, pivot shift, graft failure |
| Lee [35] | 2012 | RCT  LoE 2 | 18:19 | min 24 mo | ST, GT | **DB**: TT or AMP  **SB**: NA | EB, BIS | KT-1000, Tegner score, Lysholm score, IKDC subjective, Lachman test, pivot shift, IKDC objective |
| Misonoo [42] | 2012 | RCT | 22:22 | DB: mean 12.4 mo  SB: 12.3 mo | ST, GT | TT | EB, Cas | KT-1000, pivot shift, lysholm score, Tegner score |
| Nunez [46] | 2012 | RCT  LoE 1 | 23:29 | min 24 mo | ST, GT | **DB**: AM (TT) PL (map)  **SB**: TT | EB, BIS | IKDC subjective, graft failures |
| Ochiai [47] | 2012 | RCT | 44:40 | min 24 mo | ST, GT | TT | EB, staples | Lysholm score, pivot shift, graft failures |
| Suomalainen [54] | 2012 | RCT  LoE 1 | 45:20 | min 60 mo | ST, GT | AMP | MIS, BIS | KT-1000, Lysholm score, pivot shift, IKDC objective, graft failures, OA |
| Ahlden [5] | 2013 | RCT  LoE 1 | 48:50 | SB: median 26 mo  DB: median 26 mo | ST, GT | AMP | MIS, BIS | KT-1000, Lachman, pivot shift |
| Koken [32] | 2013 | RCT  LoE 2 | 37:30 | mean 25.8 mo | ST, GT | AMP | EB, BIS, staple | Lachman test, pivot shift, IKDC objective |
| Ventura [57] | 2013 | RCT  LoE 2 | 40:40 | min 24 mo | ST, GT | TT | RB, BIS | KT-1000, IKDC subjective, Lachman test, pivot shift |
| Xu [61] | 2014 | RCT  LoE 1 | 32:34 | mean 16.3 mo | ST, GT | map | BIS, staple, | KT-1000, Lysholm score, IKDC subjective, pivot shift, graft failures |
| Zhang [66] | 2014 | RCT | 49:45 | min 24 mo | ST, GT | TT | EB, staples, | Tegner score, Lysholm score, graft failures |
| Bohn [10] | 2015 | RCT  LoE 1 | 19:11 | mean 13 mo | ST, GT | **DB**: map  **SB**: map, TT | EB, IS, BIS | IKDC subjective, Tegner score, Lysholm score, IKDC objective, pivot shift, Lachman test, graft failures |
| Karikis [30] | 2015 | RCT  LoE 1 | 41:46 | median 64 mo | ST, GT | AMP | MIS, BIS | KT-1000, Tegner score, Lysholm score, Lachman test, pivot shift, OA |
| Koga [31] | 2015 | RCT  LoE 2 | 21:27 | SB: mean 71 mo  DB: mean 68 mo | ST | TT | EB, staple | KT-1000, Lysholm score, Lachman test, pivot shift, graft failures |
| Mohtadi [43] | 2015 | RCT  LoE 1 | 214:108 | min 24 mo | ST, GT, BPTB | **DB**: AMP  **SB**: TT | EB, NAS, | KT-1000, IKDC subjective, Tegner score, pivot shift, IKDC objective |
| Sasaki [50] | 2015 | RCT  LoE 1 | 69:67 | mean 38.9 mo | ST, GT, BPTB | **DB**: AM (TT, OI, TP) PL (TP)  **SB**: ? | EB, BIS, SD | KT-1000, tegner score, pivot shift, graft failures |
| Liu [38] | 2016 | RCT  LoE 1 | 34:32 | mean 80 mo | ST, GT | AAMP | EB, BIS, staple | pivot shift, Lachman test, graft failures |
| Mayr [39] | 2016 | RCT  LoE 1 | 28:34 | mean 26 mo | ST, GT | AMP | BCP, IS | IKDC subjective, IKDC objective, OA, graft failures |
| Mohtadi [44] | 2016 | RCT  LoE 1 | 214:108 | min 24 mo | ST, GT, BTPB | **DB**: AMP  **SB**: TT | EB, NAS | graft failures |
| Adravanti [2] | 2017 | RCT | 25:25 | min 72 mo | ST, GT | **DB**: AM (TT) PL (OI)  **SB**: TT | EB, BIS, IS, staple | KT-2000, Lysholm score, IKDC objective, OA, graft failures |
| Beyaz [8] | 2017 | RCT | 16:15 | min 96 mo | ST, GT | AMP | EB, BIS, staple | Tegner score, IKDC subjective, Lysholm score |
| Järvelä [25] | 2017 | RCT  LoE 2 | 46:24 | mean 122 mo | ST, GT | AMP | BIS, MIS | KT-1000, Lysholm score, IKDC objective, pivot shift, graft failures, OA |
| Aga [3] | 2018 | RCT  LoE 1 | 62:54 | min 24 mo | ST, GT | AAMP | EB, IS | KT-1000, Lachman test, pivot shift, graft failures |
| Mayr [40] | 2018 | RCT  LoE 1 | 25:28 | mean 63.2 mo | ST, GT | AMP | BCP, IS | IKDC objective, Lachman test, pivot shift, OA, graft failures |

*RCT* randomized controlled trial, *LoE* level of evidence, *mo* month, *min* minimum, *ST* semitendinosus, *GT* gracilis tendon, BPTB Bone-Patellar Tendon-Bone, EB endobutton, BIS bioabsorbable interference screw, MIS metallic interference screw, *CS* combination screw, *BS* bioabsorbable screw, *CP* cross pins, *IS* interference screw, *BCP* bioabsorbable cross pins, *CaS* cancellous screw, *TSK* transosseus suture knot, *RB* retro button, *NAS* non-absorbable sutures, *SD* suture disc, *SP* suture plate, *PC* post screw, *OA* osteoarthritis, *AM* anteromedial bundle, *PL* posterolateral bundle, *OI* outside in, *map* medial accessory portal, *TT* transtibial, *AMP* anteromedial portal, *MP* medial portal, *AAMP* accessorial anteromedial portal, *NA* not available, *TP* trans-portal
